# Early Childhood Development and Social Determinants

**DOI:** 10.7759/cureus.29500

**Published:** 2022-09-23

**Authors:** Akanksha Likhar, Prerna Baghel, Manoj Patil

**Affiliations:** 1 School of Epidemiology and Public Health, Datta Meghe Institute of Medical Sciences, Wardha, IND; 2 School of Public Health, Jawaharlal Nehru Medical College and Acharya Vinoba Bhave Rural Hospital, Datta Meghe Institute of Medical Sciences, Wardha, IND

**Keywords:** determinants of early childhood development, health determinants, developmental health, early childhood, social determinants

## Abstract

In human beings, the development of a child involves biological, emotional, and psychological changes that happen between birth and the conclusion of adolescence. Childhood is divided into three stages: early childhood, middle childhood, and late childhood (preadolescence). Early childhood is typically from infancy to six years of age. The methods for maintaining health and dealing with already-existing sicknesses and the social and economic settings in which children are born, grow up, live, and eventually work are referred to as the social determinants of health. Despite advances in health, child malnutrition remains a problem salutariness (severe) issue with massive human and economic resource implications. There is currently a growing corpus of research on how early development influences a child’s success later in life. From conception to two years of age, the first 1,000 days of life are becoming more well-recognized as important for the development of brain circuits that lead to linguistic, cognitive, and socio-emotional abilities, all of which are predictors of later-life labor market outcomes. The social patterning of health, sickness, and illness can be influenced by the social determinants of a child's health. This can also influence a person's overall well-being and functioning throughout their lifetime factors of a child's health, early childhood care, and development from an ecological standpoint, and as planned, a participatory approach in early childhood care and development is implemented. The social determinants of health are the elements that cause positive or negative changes in health or alter disease risks. The social determinants of health, which are different from medical treatment, can be altered by social policy. Social gradients and health equality are ideas that are related to understanding how social factors impact health.

## Introduction and background

There are some social factors and experiences that affect the health of children. Early childhood is a time frame from conception to eight years of age. The earliest years of a person's existence are thought to be the most crucial for his or her development. What happens to the child in the early years is crucial to the child's life course and developmental trajectory. Early development will be most significantly influenced by the nurturing qualities of the environment - parents, caregivers, family, and community - where children are nurtured, live, and learn. *A child is a living growing organism.* The child has a biological base and develops in a social setting. The combination of biological and environmental factors, also known as the *nature vs. nurture* factor, determines how a child develops. Early life circumstances and experiences, which are underlying socioeconomic determinants of health, have an impact on early child development. Early infancy is recognized as the most important developmental years of a person's life. The early experiences of a child have a substantial impact on his or her developmental trajectory and life fate [1]. The nurturing aspects concerning the environment in which a child grows up, resides, and gains knowledge influence his or her early development. A child is born into a biological family and grows up in a social setting. The combination of biological and environmental forces, commonly referred to as nature, is crucial for development [1]. Social determinants are characteristics of people's environments to which they are *exposed* and that might influence their long-term developmental and health consequences [1]. Social determinants impact people in a variety of ways, interact with one another, and cover a wide spectrum of attributes that are not dependent on biology or genetics, including housing circumstances; interpersonal interactions among children, parents, and peers; family sociodemographics; day-care and school learning settings; fit to the outdoor areas; safety in the neighborhood; and the sociopolitical setting [2].

Experiences throughout this period, and even before birth, have a long-term impact on the health, education, and economic prospects of a child. Experiences in the first six years can become physiologically imprinted and impact outcomes throughout life, both positively and negatively [2]. Disruptions during this time can have a major influence on behavior, learning, and adult health consequences. Fortunately, acting early, and frequently, can have a significant impact on promoting favorable outcomes and minimizing or mitigating the impact of negative childhood experiences and events [3]. Early childhood development programs (such as education and care, family support, and poverty reduction) produce long-term benefits that outweigh the initial expenditure many times over [4]. Early childhood development is a health determinant: early childhood health, well-being, and learning ability are all affected by development throughout life.

Programs and services for children with special needs: Early childhood development

Early childhood investment is a powerful economic approach that yields several times the initial investment above the way based on individual existence. Early Childhood Development programs aim to improve the quality of human capital or people's abilities to participate in society and the workforce. Early Childhood Development programs not only build competencies and abilities that will be never restricted to percipient earn but also encompass physical, social, and emotional improvements based on the determinants of well-being across the direction of a person's life. As a result, Early Childhood Development programs that combine and integrate well-being-advance plans (e.g., excellent diet and vaccination) with nurturing, involvement, care, stimulation, and protection have the potential to provide long-term benefits in physical, social, emotional, linguistic, and cognitive development [5].

## Review

Data source

We conducted a search of electronic databases with a specific goal in mind to find literature, a quest for reliable summaries, and various sources, including books, that have detailed conceptual and theoretical studies, handbooks, and gray literature. We took a look at what medical and social experts have been working on to discover the parameters that allow children to achieve optimal health and development. A literature search of various journal indexes such as PubMed, Embase, Google Scholar, and others was done systematically.

Search Terms

Major search terms were social determinants, early childhood, developmental health, and determinants of early childhood development.

Selection Criteria

Inclusion criteria for the study were all children aged between conception and eight years as well as children living with parents. Exclusion criteria included case-control and cohort studies among children aged more than eight years, adolescents, and older age groups.

Discussion

Determinants of Early Childhood Development

Early childhood, sometimes known as *the early years*, is the most essential developmental stage of life, during which critical advances are made in the physical, social, cognitive, emotional, family environment, and linguistic domains.

Physical Development

Genetics plays a role in physical development. Genes control the number of hormones produced, which affect the pace of advancement. Hormones are substances generated by glands and released into the blood circulation [6]. Growth hormone is produced from birth and has an impact on practically every element of the body's development. Growth hormone deficiency causes slower growth in children; however, its supplements can help to boost growth when needed. Physical development is one of the many aspects of newborn and toddler development [6]. It has to deal with the development and growth of the brain, muscles, and senses. Babies, as their bodily senses of sight, touch, smell, sound, and taste grow, learn information about the world [7]. They can hear long before from the moment they are born, like staring at people's faces, and seek out intriguing things to gaze at. A newborn may recognize his or her mother's aroma and voice within days after birth [7]. Infants are aware of their environment from birth, and as they begin to explore with their senses, their potential to grow, develop, and learn accelerates [7].

Growth and Motor Development

According to research, children's growth is high during the initial two years and then decreases throughout the early stages of childhood between two and six years of age. On average, a child grows 2 to 3 inches taller and gains over 5 pounds every year [8,9]. Generally, a six-year-old child weighs 45 pounds and is 46 inches tall. The height and rate of growth of children are highly related to those of their parents. However, there are several other factors as well. Surprisingly, in a society, a few family trees are significantly giant than others. Babies' appetite is likely to drop between two and six years of age [10]. Some argue that young children's dislike of unusual foods is evolutionarily adaptive because it urges them to consume familiar and safe foods instead of uncommon and highly harmful foods. Picky eating becomes less prevalent as a child grows older, but for many youngsters, it remains a persistent issue that lasts for years. Picky eating appears to be a recurring feature in individuals [11]. This example shows how developmental domains interact dynamically, with temperament, an emotional element, impacting food and influencing physical growth. Early childhood meals must be prepared in certain ways, and children must exhibit strong likes and dislikes, as well as throw feeding tantrums. Young children need the same foods as adults for a healthy diet. Although most youngsters in wealthy countries get enough calories, they are frequently deficient in vitamins and minerals [11]. Foods abundant in calcium, iron, and zinc are commonly neglected in favor of less healthy choices. Although sweetened cereals include numerous vitamins and minerals, the glucose content increases the probability of primary obesity along with added well-being complications in children, including stoutness. In one research, cereals for children were compared with those offered to adults. According to the survey, almost all cereals make up two-thirds of children who have not met US nutrition guidelines for school meals. Roughly half to one-third of all diets achieved the milk criteria, 13% met the whole grain recommendations, and 7% met the dark vegetable recommendations. As we cannot afford full-time child care, it is likely that the diets of the enrolled children will not be able to meet the official nutritional criteria [12]. Deficiencies of vitamins A, B, D, and K, as well as iron and calcium, in preschoolers are all included, and these deficiencies encounter detrimental effects on children's development worldwide. Many children in underdeveloped nations are malnourished chronically [13]. Children's growth is jeopardized by poor nutrition. During the drought, children's food consumption plummeted and primary school students gained just half as much weight as required. Malnutrition has a variety of effects on development and not just growth. Children who are malnourished have cognitive difficulties, as well as problems with motivation, curiosity, and the capacity to engage with their surroundings [13]. During the drought in early childhood, malnutrition causes long-term deficiencies. Malnutrition is a worldwide issue, not only in impoverished nations. Because of socioeconomic considerations, a large number of children in industrialized nations, such as the United States, are underprivileged by foods that encourage good growth. Low-income households may struggle to provide a diverse choice of foods for their children's healthy growth. Table 1 shows the characteristics of the included studies.

**Table 1:** Characteristics of included studies. PA, positive affectivity

Author name	Year	Study design	Study participants/age	Outcome
Hertzman [3]	2000	Observational study	*n* = 118	Children with poor motor skills were less active than those with better-developed motor skills.
Black et al. [9]	2016	Intervention study	Children aged <3 years	This paper, the first in a three-part series on early childhood development, examines the recent scientific progress and global commitments to early childhood development.
Donnelly et al. [12]	2016	Cross-sectional, longitudinal, acute, or intervention trials	Children aged 5–13 years	PA has a positive influence on cognition as well as brain structure and function.

Motor Development

A major development in early life is the refining of motor abilities that require hand-eye coordination and little movements and those that engage the body's primary muscles [13]. Children between three and six years of age make significant progress in running and jumping, which are examples of gross motor abilities. Coordination improves in children, and the sensory and motor centers of the brain’s abilities mature. Both may now take part more aggressively in more difficult games such as running, leaping, and climbing. Poor agileness is linked to socioeconomic disadvantage, and other elements like physical and cognitive growth are possibly affected due to poor nutrition and less opportunity to practice motor skills in the environment [13]. Low-income neighborhoods may lack the facilities to provide a playground, amusement centers, and security, thereby encouraging children to play outside. Jumping, running, and riding tricycles, pedal vehicles, and other riding toys are all good ways for young children to exercise their large motor skills. Complex motions, such as those required to ride a bicycle, are arduous for small children to master as they require coordination of many limbs, stability, and other skills. By five years of age, children can toss, climb a ladder, catch and kick a ball, and pedal a three-wheeler [14]. Advancements in terms of gross motor skills aid children in moving around and gaining sensation of power over habitat, while dexterity enables little kids to accept that they are given responsibility for their care.

Family Environments

From the moment they are born, children engage with their families. Family plays a crucial part in energizing, supporting, and nurturing children. These characteristics, in turn, depend on a family's capacity to invest in raising children (which is greatly influenced by affluence), parenting style, and propensity to establish a flexible and adaptable linguistic environment (highly impacted by parental educational levels) [15]. As risk and protective variables, familial characteristics may play a role that impacts children's growth in both good and bad ways. Whereas the meaning of *family* varies according to sociocultural context and historical situations (e.g., direct family, extended family, community, or clan), we describe familial ties with a child and the possibility of intimate bonds with the child. We believe the feature of *ideal early childhood environments*, which helps children's growth and transcends any specific *family definition*, is more important than defining the characteristics of a *family* or *household* [15]. Researchers in North America who began working on this project more than three decades ago noticed that children from low-income households did not develop a similar level of linguistics as children from high-income homes in terms of cognitive abilities. Poverty, as was thought, places children in danger due to a scarcity of resources, for example, inadequate nutrition, such as calcium, vitamin, and protein deficiencies. All of these nutrients are necessary to ensure optimal physical and cognitive growth in children. Therefore, according to recent studies, children from poor families have worse academic success, social skills, and cognitive functioning than children from affluent families [16]. Other essential social characteristics of a child's environment have also been connected to healthy early child development in these studies as the most important factors. A recent study has discovered several factors that influence early infant development, including sufficient maternalistic nutrition, parental stress and depression, parenting methods, mental and physical health, unemployment, earnings, home circumstances, and the standard of living in the neighborhood. Both child’s health and academic attainment are affected by these factors. Penury has a negative impact on cognitive and linguistic development.

While relatively well-off families have benefited from the recent rapid societal transformation. Many less well-off families have suffered as the increased demands of modern life and parenting may be stressful for them, with detrimental effects on their children. The more advantaged a family outset, the greater improvement ability to leverage and expand on the enhanced prospects available, and the gap between them and others who are incapable progressively grows [17]. Again, the number of families with various complicated demands looks to be on the rise. Such families are frequently confronted with a wide range of outside pressures, including homelessness, poverty, and social isolation, and their parents may be coping with their abuse and trauma. Poor housing, especially homelessness, has a severe impact on children's health and well-being [18]. Housing infrastructure interventions do not appear to eliminate housing disparities in these areas, necessitating a multilevel and multifaceted *ecological* approach.

Poverty and Cognitive/Language Development

Living in poverty has long been linked to poor health, development, school performance, and accomplishment in children as well as poor adult health [18]. There were considerable discrepancies in the diversity of children’s linguistic environments from a poor economic background household compared with their more economically advantaged peers, resulting in the children having worse learning of a language [18]. One of the most basic needs of children, such as safe housing, nutritious food, and excellent child care, can be influenced by poverty. Brooks-Gunn, an American psychologist, investigated the effects of household earnings on the behavior and IQ of children and discovered that mental assets such as nuclear family support and mother education are important. The findings of youngsters were impacted by their mental health. Risk factors for early child development include inadequate maternal education, lack of mental well-being, and scarcity of family networks [19].

Emotional Development

A mix of dynamical systems theory and functionalist theory has been a nearly new viewpoint on children's emotional development: a lack of family networks, mental illness in the mother, and a lack of education have all been recognized as risk factors. These are all illustrations of dynamic interactions with many emotion-related elements that alter over time as the child develops and in response to shifting contextual interactions [20]. Emotional development alters by various social and cultural factors aspects, to name a few backgrounds. Sentimental abilities emerge as a result of a growth process in which a particular talent that is nurtured emerges individually with various periods [21]. Early on, emotion knowledge is more definite in children, with an increased emphasis on visible characteristics. Emotion knowledge is more definite in early childhood, with a greater emphasis on observable aspects. Elementary school students improve their abilities to self-report emotions and use language to describe emotional circumstances [22]. Children's assumptions about what others are experiencing get more complex as they grow older, including providing not just data but also knowledge from previous exposure to the past. Early on, children are also superior at comprehending and expressing complicated feelings like pride, shame, and humiliation [23]. Adolescents experience a higher sense of desire and potential when they enter adolescence due to a combination of similar, ethical, and other stimuli [24,25].

## Conclusions

Researchers and policymakers alike recognize the essential relevance of the earliest years of life. This study also shows that the consequences of early childhood development enhance lifetime health factors. The goal of this study is to provide a comprehensive assessment by researching the endless socioeconomic determinants of early childhood development. There is a complex relationship between the numerous levels at which social factors play a role to operate. This study outlines a few fundamental ideas that can help both developed and developing nations to increase the developmental outcomes of children in their early years of life and during important changes such as school enrollment. While it is critical to define the broad principles and processes of social determinants, scholars must continue to strive for a better understanding of how unique cultural and geographical circumstances influence the result of the interplay of social factors.
